# Supplementary material for: Structural basis for lamin assembly at the molecular level
Source: Nat Commun. 2019 Aug 21;10:3757. doi: 10.1038/s41467-019-11684-x (PMC6704074; doi:10.1038/s41467-019-11684-x)
Supplement: Supplementary file 1 — Supplementary Information [file 41467_2019_11684_MOESM1_ESM.pdf]

Supplementary Information

Structural basis for lamin assembly at the molecular level

J. Ahn, I. Jo et al.

Supplementary Table 1. X-ray diffraction and refinement statistics.

**Data collection**	**Lamin 300 fragment (residues 1-300)**
Beamline	Pohang Accelerator Laboratory Beamline 5C
Wavelength (Å)	0.9795
Space group	$P2_12_12$
Cell dimensions	
a, *b*, *c* (Å)	231.3, 85.0, 92.3
α, β, γ (°)	90, 90, 90
Resolution (Å)	50-3.2 (3.26-3.21)
No. of reflections	40335
R_{merge}	0.11 (0.37)
R_{pim}	0.04 (0.21)
$I/\sigma I$	11.5 (3.11)
Completeness (%)	93.5 (80.8)
Redundancy	5.7 (3.4)
**Refinement**	
Resolution (Å)	46.2-3.20 (3.26-3.21)
No. reflections	40280
R_{work}/R_{free}	0.207/0.260
Total no. of atoms	7161
No. ligands	0
No. water molecules	0
Wilson B-factor (Å^2)	39.3
Average B (Å^2)	46.0
R.M.S deviations	
Bond lengths (Å)	0.002
Bond angles (°)	0.453
Ramachandran plot	
Favoured (%)	99.09
Allowed (%)	0.93
Outliers (%)	0.0
PDB ID	6JLB

* Values in parentheses are for the highest resolution shell.

** $R_{merge} = \Sigma_{hkl}\Sigma_i|I_i(hkl) - [I(hkl)]|/\Sigma_{hkl}\Sigma_i I_i(hkl)$, where $I_i(hkl)$ is the intensity of the *i*th observation of reflection *hkl* and [I(*hkl*)] is the average intensity of *i* observations.
*** $R_{p.i.m.} = \Sigma_{hkl}\,(1/(n-1))^{1/2}\,\Sigma_i|I_i(hkl) - [I(hkl)]|/\Sigma_{hkl}\Sigma_i I_i(hkl)$. R_{pim} is the precision-indicating (multiplicity-weighted) R_{merge}.
****R_{free} calculated for a random set of 7.8% of reflections not used in the refinement.

Supplementary Table 2. Summary of the 52 kDa bands (52 kDa #1 and 52 kDa #2) from Supplementary Data 1 and supports Fig. 5b.

residue number	# observed peptides	# CL-linked peptides	proportion of cross-liking ($\hat{p}$)	standard error of p
K32	38	0	0	0
K78	ND	ND	ND	ND
K90	69	4	0.06	0.028
K97	73	0	0	0
K108	136	14	0.11	0.026
K114	49	0	0	0
K117	61	0	0	0
K122	61	0	0	0
K123	61	0	0	0
K135	26	0	0	0
K144	29	5	0.17	0.070
K155	29	2	0.07	0.047
K171	25	8	0.32	0.093
K180	75	9	0.12	0.037
K181	76	9	0.12	0.037
K201	12	0	0	0
K208	28	3	0.11	0.058
K218	8	0	0	0
K233	79	7	0.09	0.032
K260	81	2	0.02	0.017
K261	81	11	0.14	0.038
K265	207	19	0.09	0.020
K270	38	5	0.13	0.055

* ND = not detected

** standard error of $p = \sqrt{\frac{\hat{p} * (1-\hat{p})}{\text{number of obserbed peptides}}}$

Supplementary Table 3. Oligonucleotide primer sequences of human lamin fragments

lamin fragments	forward primers (5'→3')	reverse primers (5'→3')
lamin 1-125	ggcgaattcatggagaccccgtcccag	ggcaagcttttaaccctccttcttggttatg
lamin 1-300	ggcgaattcatggagaccccgtcccag	ggcaagcttttagtcgatgcggatgcg
lamin 175-300	ggcgaattcatggccctaggtgaggcc	ggcaagcttttagtcgatgcggatgcg
lamin 250-400	ggcccatggcccagcatgaggac	ggcctcgagctagccacggctgcgctg
lamin 286-400	ggcgaattctgggggctgcccacgagg	ggcctcgagctagccacggctgcgctg
lamin 406-567	ggcgaattctcatcccagacacagggtg	gccaagcttttagtggtggtgatggagcag
lamin Y45C	cgcttggcggtctgcatcgaccgtgtg	cacacggtcgatgcagaccgccaagcg
lamin L59R	gagaacgcagggcggcgccttcgcatcaccgag	gatgcgaaggcgccgccctgcgttctccgtttc
lamin A146C	ctgaactccaaggagtgcgcactgagcactgc	gcagtgctcagtgcgcactccttggagttcag
lamin R388C	aggaggagaggctatgcctgtccccc	gggggacaggcatagcctctcctcct

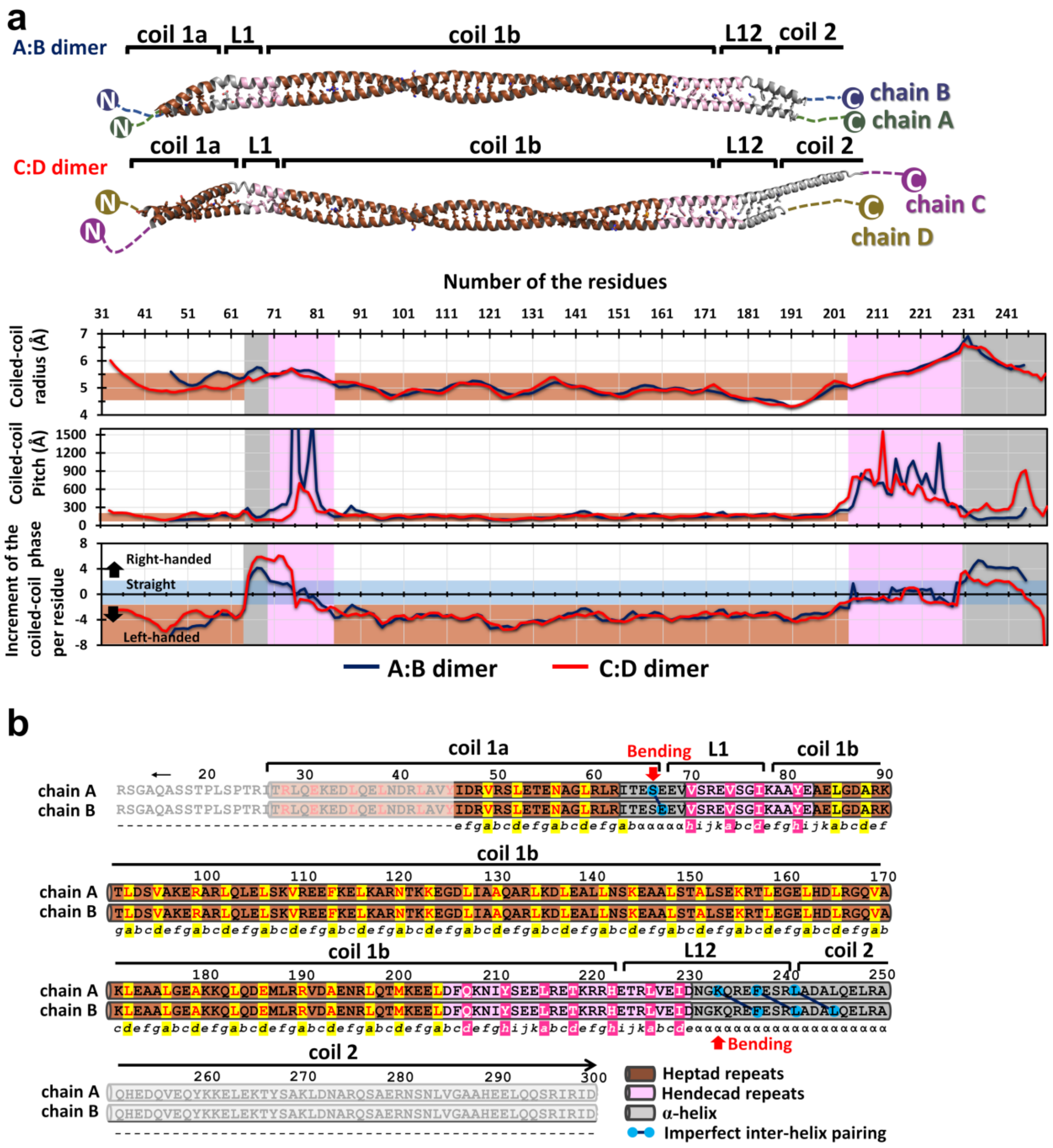

Supplementary Fig. 1. Structural and geometrical analyses of the A:B and C:D helical dimers

a Structure and geometry of coiled-coil were analysed using the program TWISTER [1]. The heptad repeat periodicity is in brown, with the coiled-coil (CC) pitch ranging from 100-200 Å. The hendecad repeat periodicity is shown in pink with more than 300 Å of the CC pitch. Regions lacking regular inter-helical interaction are shown in grey. Graphs representing the CC radius, CC pitch, and the increment of the coiled phase per residue are displayed.

b Structure-based superhelicity and sequence periodicity of the A:B dimer in the lamin A/C fragment (residues 1-300). The colour schemes are shown as in Fig. 2a. Structure-based analysis of the sequence periodicity of the heptad (shaded in brown or yellow) or hendecad (shaded in pink or magenta) repeats. The phases of the heptad (abcdefg) and hendecad (abcdefghijk) repeats are described below the sequences. The hydrophobic residues (a, d, and h) are highlighted by shadowing. α indicates an α-helix that does not follow the heptad nor the hendecad rules. The bends in the α-helices are indicated by red arrows. The mismatched interaction between two α-helices is highlighted with linked blue circles. The disordered regions are masked with semi-transparent boxes.

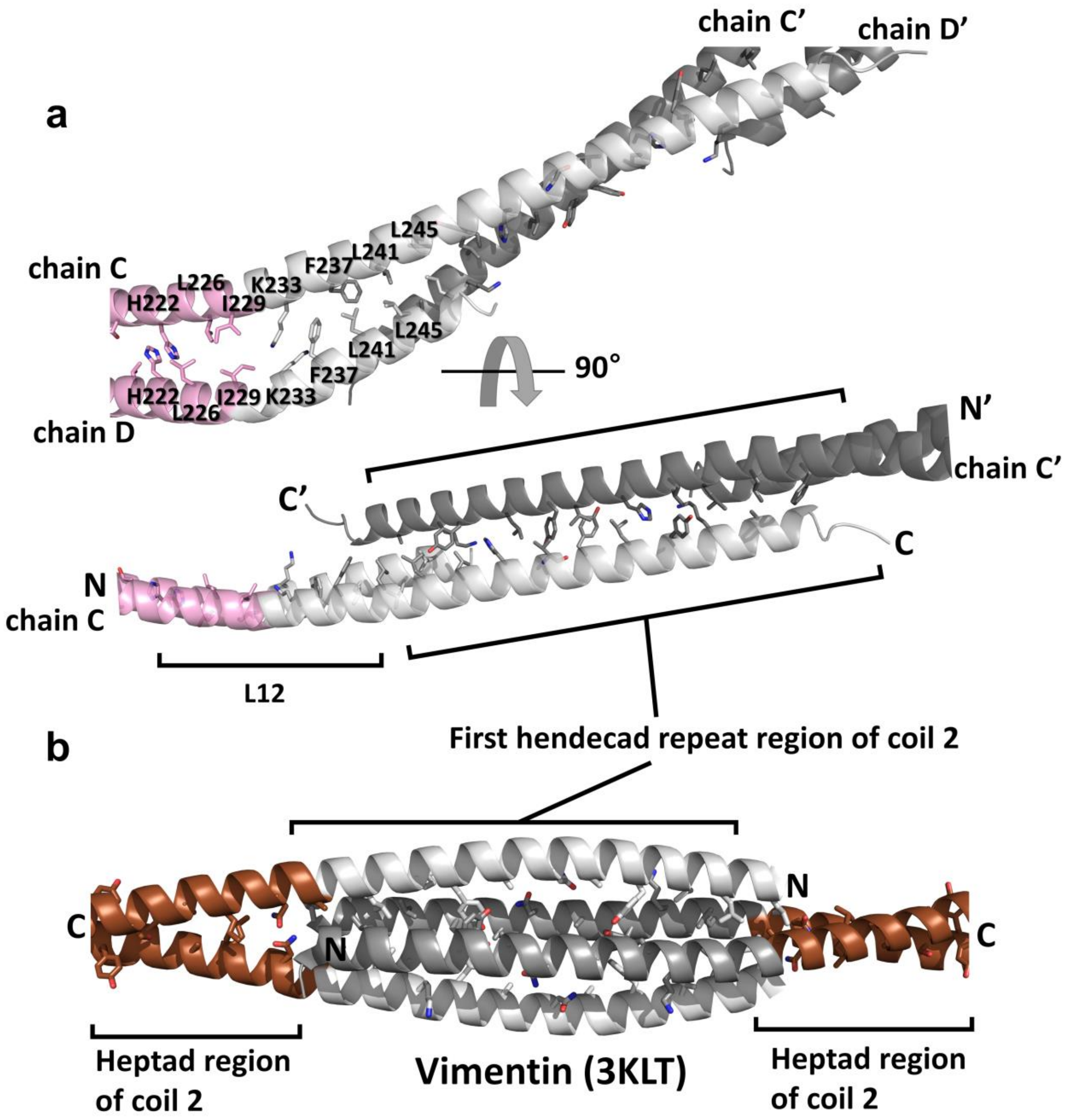

Supplementary Fig. 2. Anti-parallel α-helical interactions of lamin and vimentin found in their crystal structures

a The orthogonal view of the coil 2 region in the C:D dimer of the lamin 300 structure in the crystal. Chains C and D (pink or grey) and their symmetry-related chains C' and D' (dark grey) are displayed in ribbon representations with inter-helical hydrophobic residues (stick representations). The first hendecad repeat region (residues 241-277) of coil 2 is involved in the packing interactions.

b The coil 2 region of the vimentin fragment (PDB code: 3KLT[2]) in the crystal. Similar to that of lamin A/C, the first hendecad repeat region (residues 265-301, corresponding to residues 241-277 in lamin A/C) of coil 2 is involved in the packing interactions.

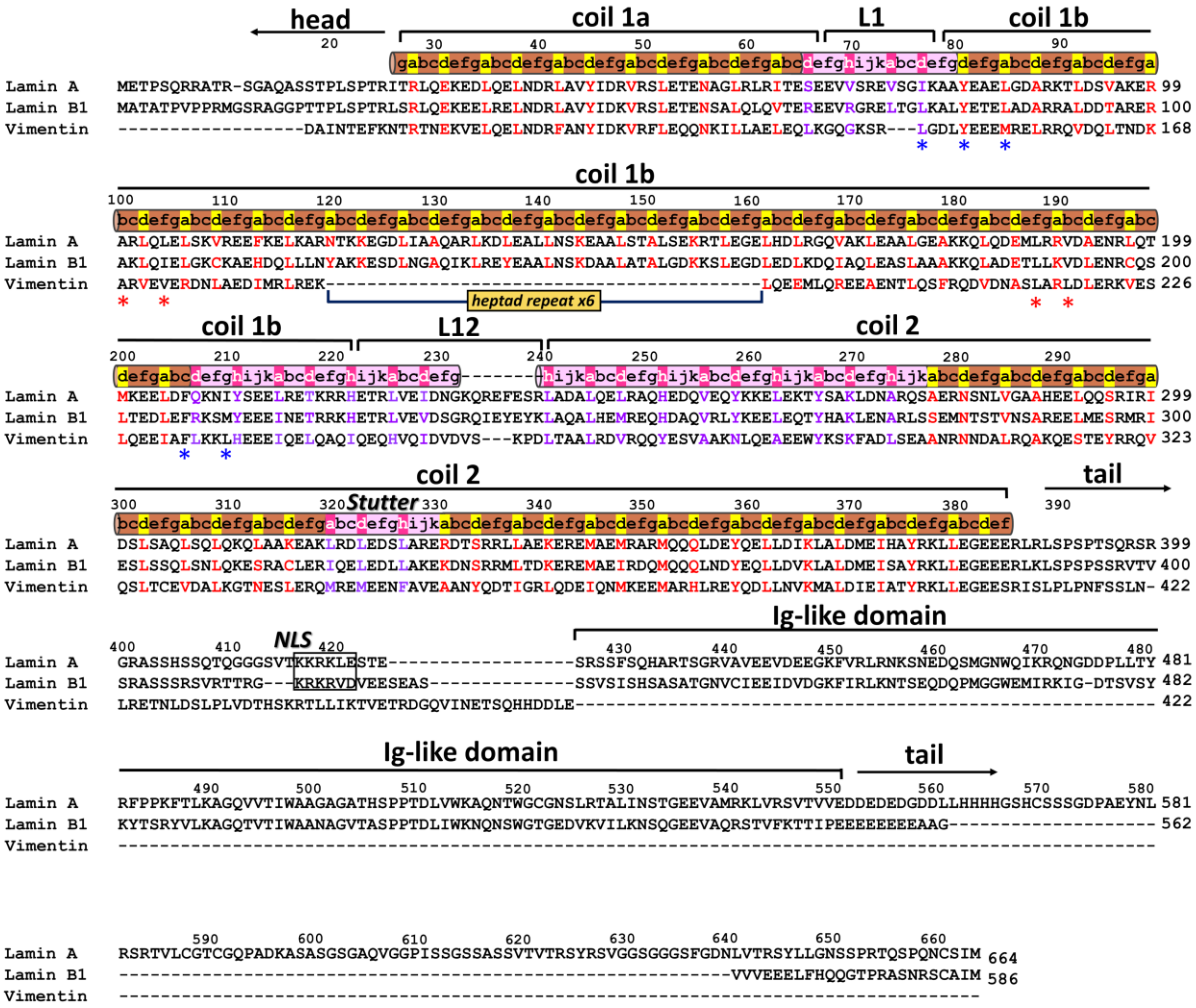

Supplementary Fig. 3. Sequence comparison of lamin A, lamin B1, and vimentin

The domains and subdomains are indicated. The phases in the heptad (brown) or hendecad (pink) repeat, which were previously predicted [3, 4], are displayed above the sequences. Gaps in vimentin coil 1b were generated based on structural comparison. Note that the nuclear localization signal (NLS) and Ig-like domains are found only in lamins. The conserved residues associated with the tetramer interaction between lamin and vimentin are marked with asterisks.

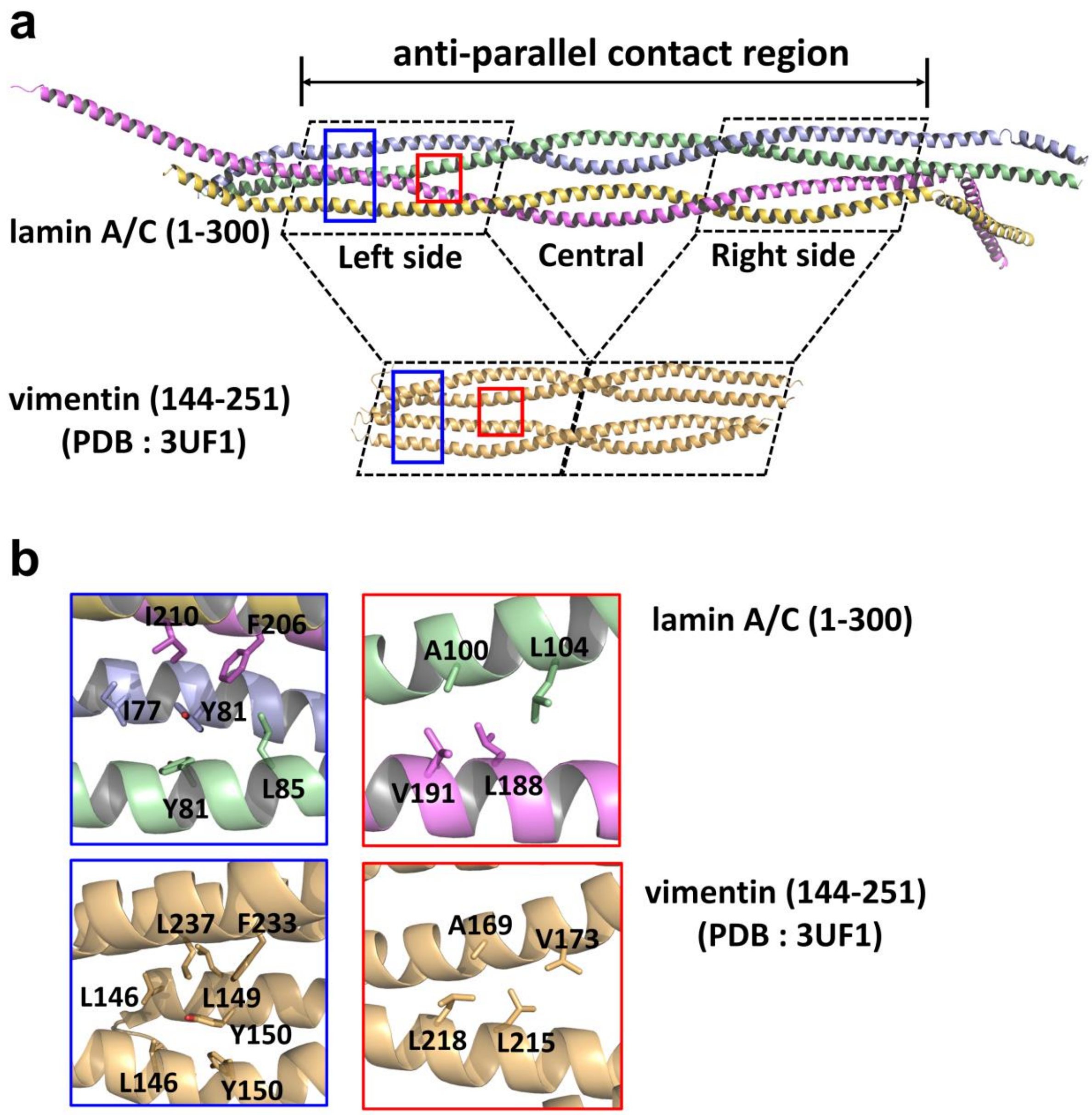

Supplementary Fig. 4. Structural comparison of the anti-parallel contact regions of the lamin 300 fragment (this study, residues 75-213) and vimentin coil 1b (PDB code: 3UF1[5], residues 144-251)

a Left and right-side parts of the vimentin structure (bottom) are directly joined to the corresponding regions in the lamin 300 fragment (top).

b Interface regions forming the tetramer (boxed regions in A) are enlarged (top for lamin, bottom for vimentin).

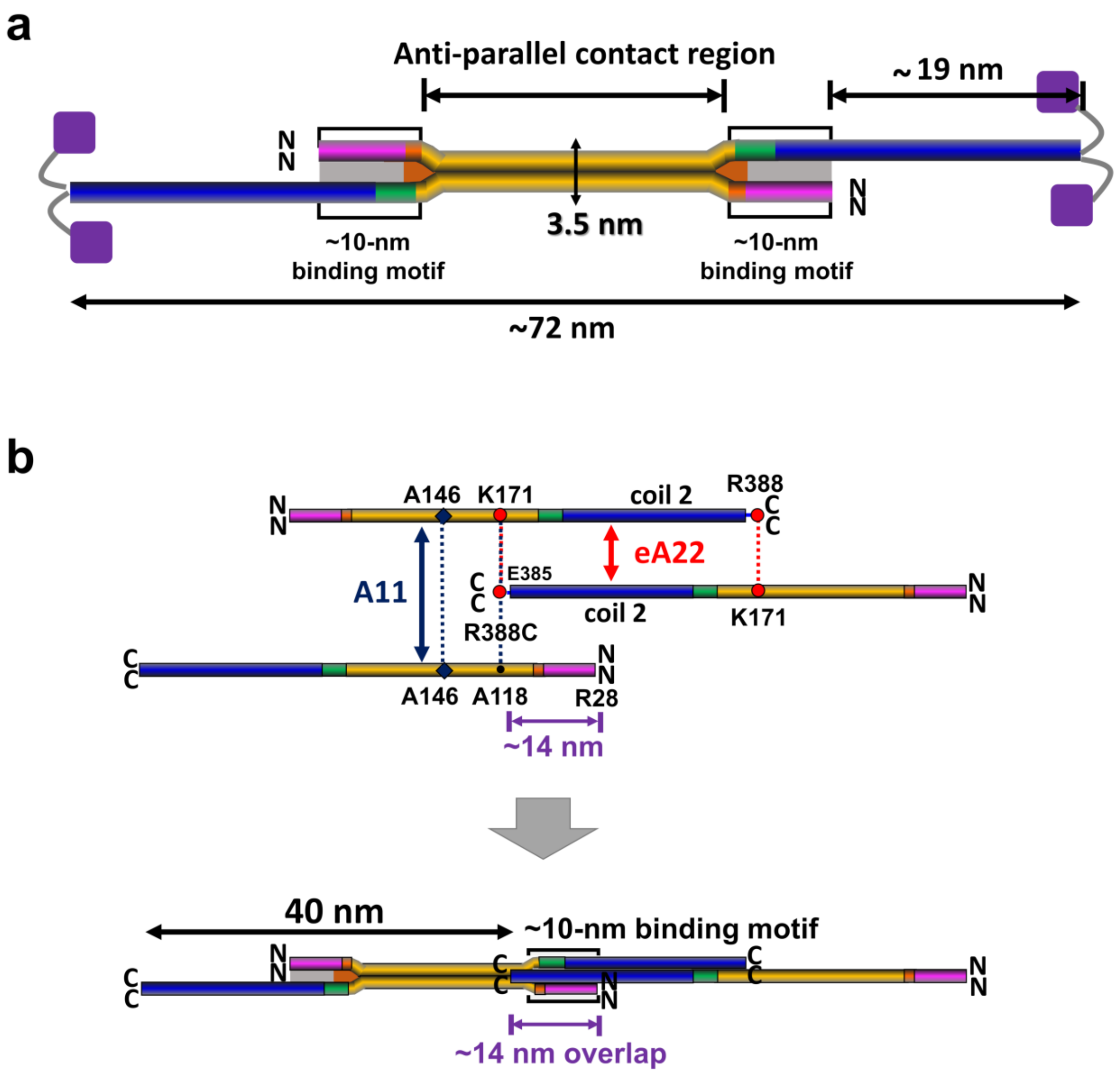

Supplementary Fig. 5. Construction of a full-length model for lamin A/C

a Schematic drawings of the full-length tetramer model based on the tetramer structure of the lamin 300 fragment. Black boxes indicate the 10-nm-long site in the anti-parallel contact region of the lamin tetramer. The lengths are indicated by double-headed arrows.

b The two interactions are combined in three coiled-coil dimers. The A11 interactions are indicated by blue lines, and the eA22 interactions by the red lines (top). The combined interactions result in ~14-nm-long overlapping of three dimers consisting of 6 α-helices in the intersection, and a ~40-nm-long interval between the C-terminal ends of two coiled-coil

dimers in the same direction (bottom). The ~10-nm binding motif in the A11 tetramer, described in Fig. 6, is indicated.

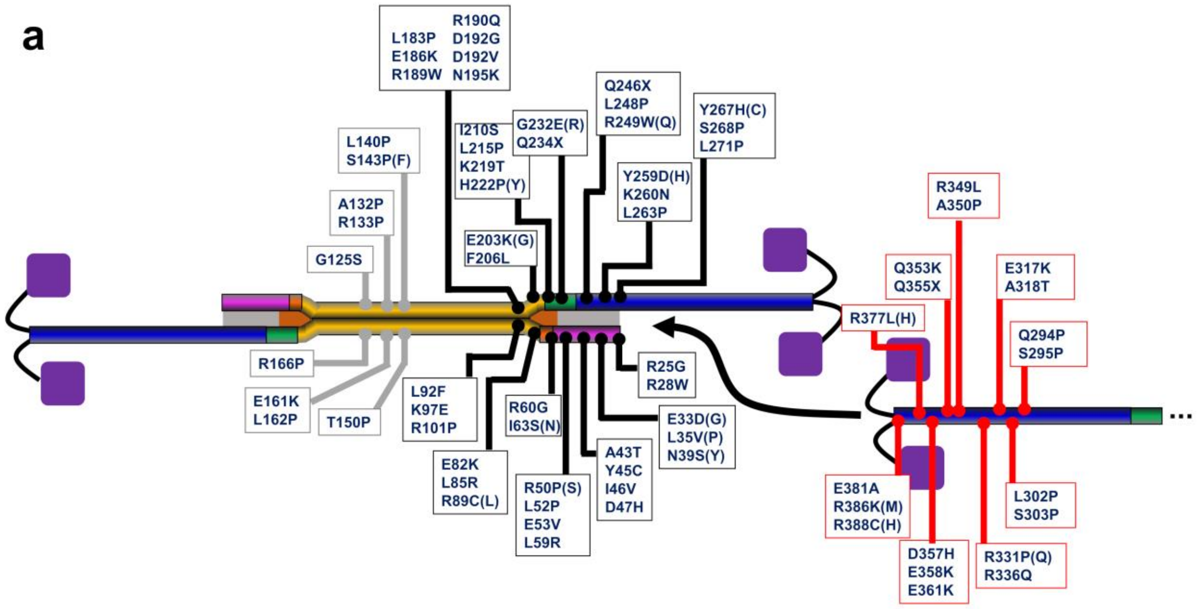

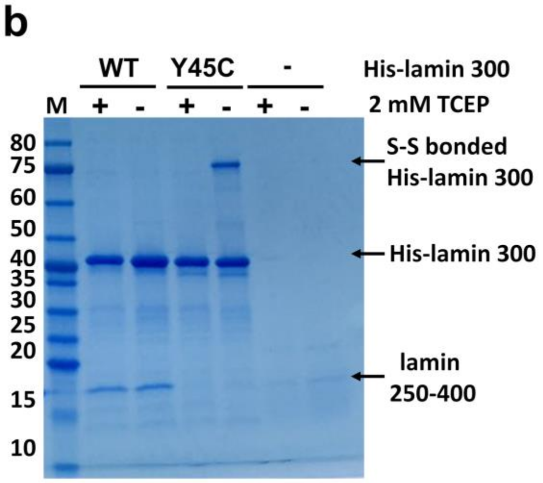

Supplementary Fig. 6. Association of the lamin assembly and laminopathies with preferential involvement of skeletal and cardiac muscle on the lamin rod domain.

a Mapping of mutations associated with skeletal and cardiac muscular related laminopathies on the lamin rod domain, based on our assembly model [6, 7]. For detailed information, see the mutations in red of Fig. 6 in reference [6]. Black dots are in the 10-nm-long site, red dots are in the C-terminal coil 2, and grey dots are in the central part of the anti-parallel contact region.

b *In vitro* binding assay using the wild type lamin 300 fragment or Y45C mutant protein-bound Ni-NTA resin. A lamin fragment (residues 250-400) was incubated on resin pre-equilibrated with a high-salt buffer containing 20 mM Tris-HCl (pH 8.0) and 150 mM NaCl. The buffer did not contain reducing agents for non-reducing SDS-PAGE, while 2 mM TCEP

was added to the buffer to break the disulfide bonds of the sample. Molecular weights (kDa) of the marker (M) are labelled on the left

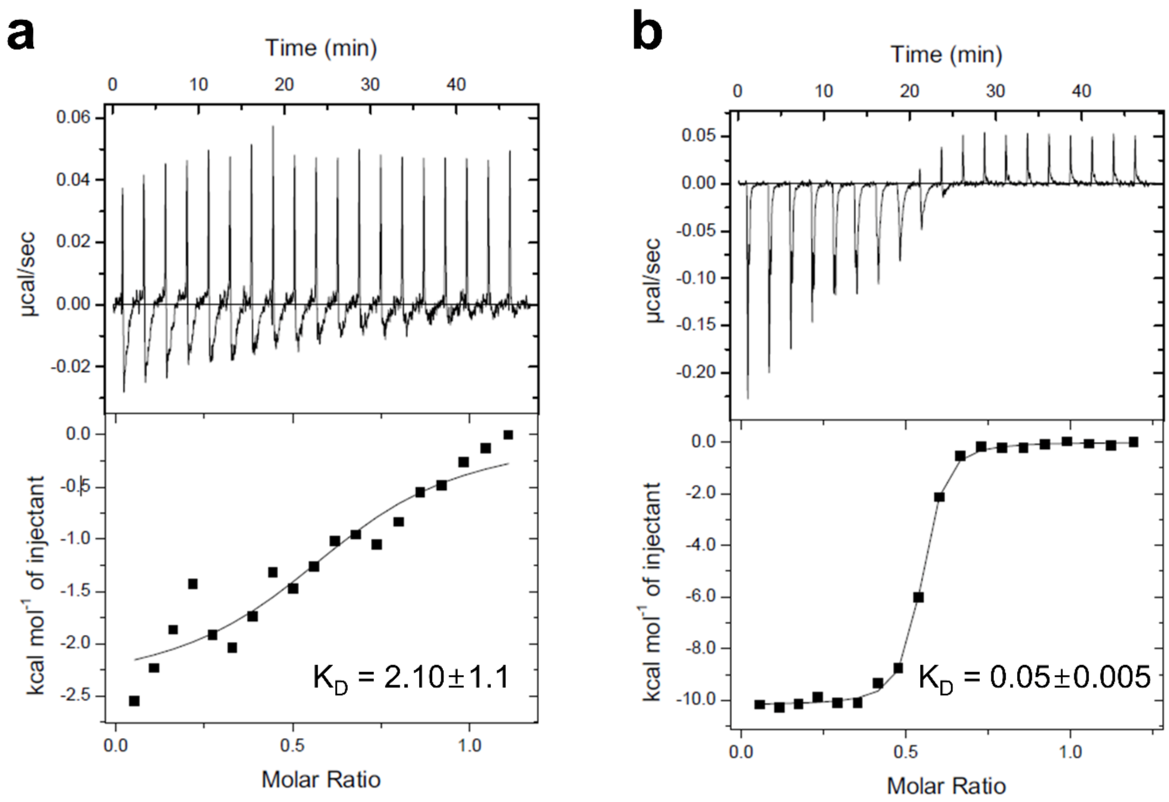

Supplementary Fig. 7. Isothermal titration calorimetry (ITC) analysis for the binding of lamin 300 fragments (WT; a and L59R; b) to the coil 2 fragment (residues 250-400). The top panels represent the raw data plots as a series of peaks corresponding to the heat change (µcal/s) resulting from titration of lamin 300 fragment (20 µM; 370 µL) with 19 injections of the coil 2 fragment (160 µM; 2 µL per one injection). The bottom panels show the integrated heat pulses in the top panels.

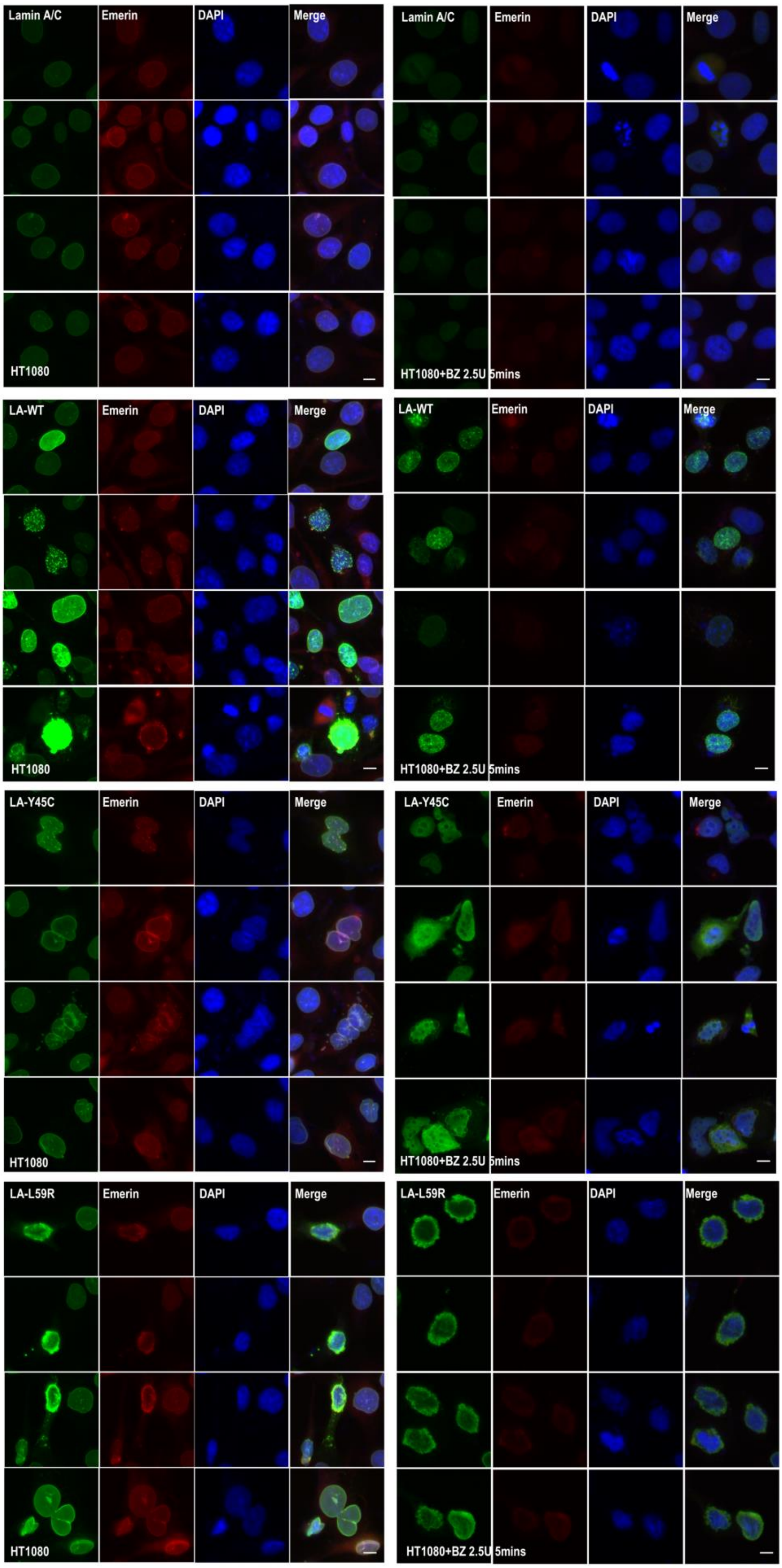

Lamin A/C
Emerin
DAPI
Merge
HT1080
Lamin A/C
Emerin
DAPI
Merge
HT1080+BZ 2.5U 5mins
LA-WT
Emerin
DAPI
Merge
HT1080
LA-WT
Emerin
DAPI
Merge
HT1080+BZ 2.5U 5mins
LA-Y45C
Emerin
DAPI
Merge
HT1080
LA-Y45C
Emerin
DAPI
Merge
HT1080+BZ 2.5U 5mins
LA-L59R
Emerin
DAPI
Merge
HT1080
LA-L59R
Emerin
DAPI
Merge
HT1080+BZ 2.5U 5mins

Supplementary Fig. 8. Immunofluorescence assay for visualization of nuclear shapes and distribution of the Y45C and L59R mutants of lamin A/C

Nuclear morphology was examined using fluorescence confocal microscopy after transfection of wild type or the Y45C or L59R mutant form of lamin A/C into HT1080 cells with or without benzonase. Cells were transfected with the indicated vectors for 24 h and treated 2.5 U/ml benzonase for 5 min during permeabilization with 0.1% (v/v) Triton X-100. For visualization of the nuclear membrane, cells were stained with lamin A/C (green), emerin (red) and DAPI for DNA (blue). Scale bar: 10 μm.

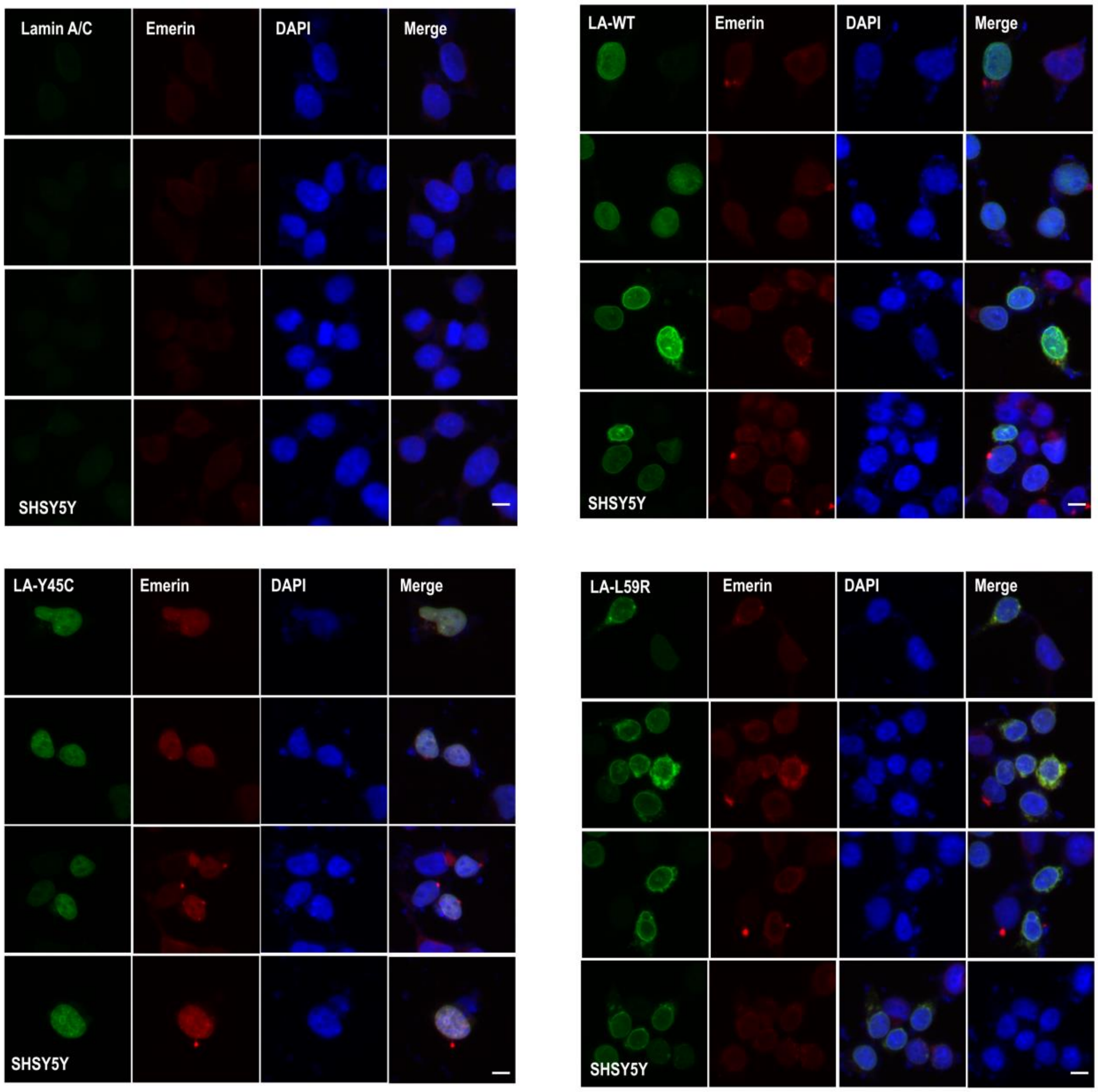

Supplementary Fig. 9. Nuclear shape and distribution of lamin A/C in the human neuroblastoma cell line (SHSY5Y) transfected with Y45C or L59R mutant lamin A/C

Nuclear morphology was examined using fluorescence confocal microscopy after transfection of wild type or the Y45C or L59R mutant form of lamin A/C into SHSY5Y cells. For visualization of the nuclear membrane, the cells were stained with lamin A/C (green), emerin (red) and DAPI for DNA (blue). Scale bar: 10 μm.

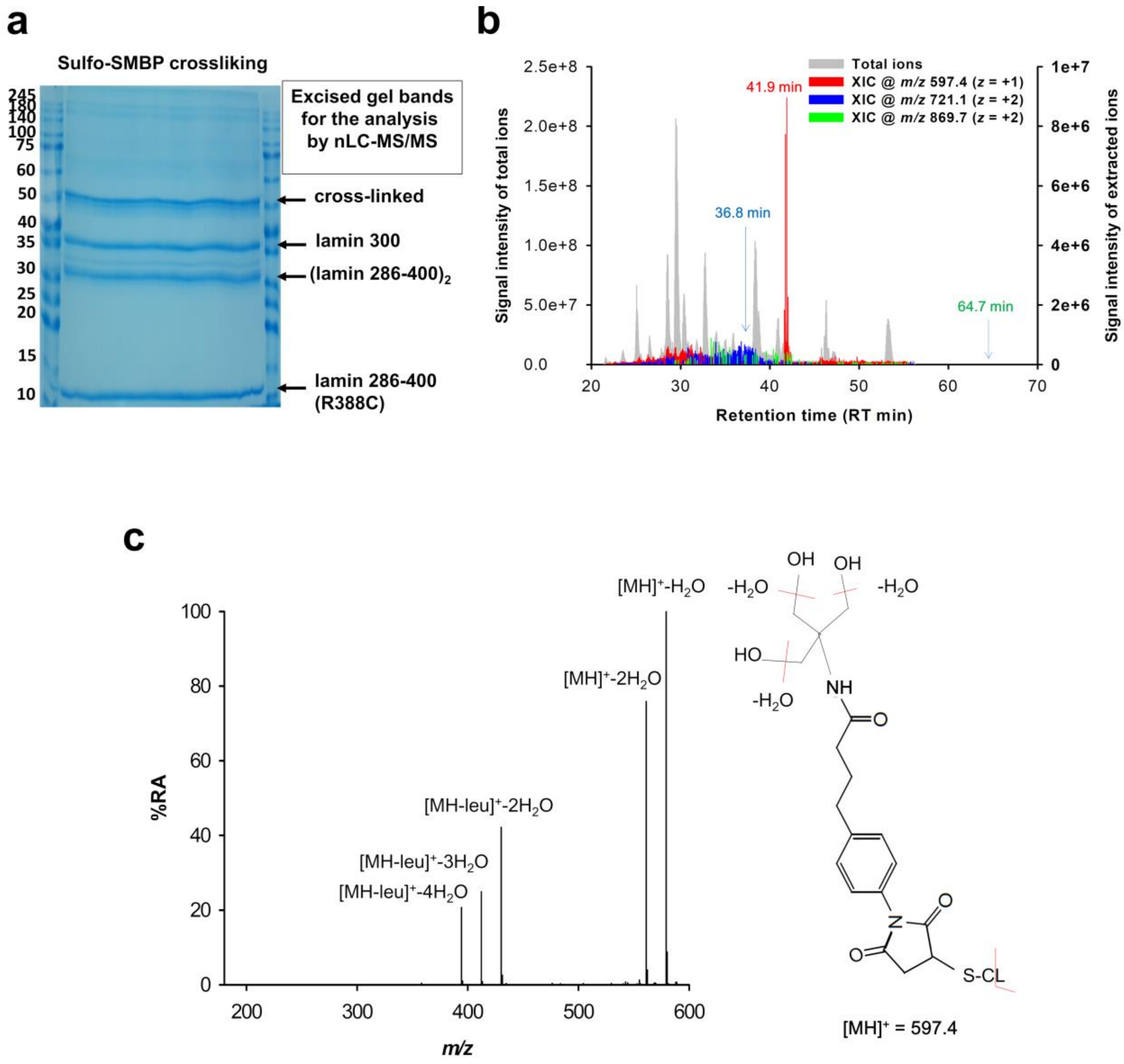

Supplementary Fig. 10. Extracted ion chromatogram (XIC) and a tandem mass spectrum of Tris-blocked cross-linker bound to Cys388-Leu389 dipeptide in the coil 2 (286-400) R388C mutant protein

a Excised gel bands for the analysis by nLC-MS/MS. Protein bands of the lamin 300 fragment and the cross-linked sample are labelled. To block the unreacted chemical cross-linker, excess cysteine was added. The major bands of 'cross-linked' (52 kDa), lamin 300 (38 kDa), (lamin 286-400 R388C)$_2$ (30 kDa), and lamin 286-400 R388C (14 kDa) were excised and subjected to the subsequent MS/MS analysis.

b Total ion (left y-axis) and extracted ion (right y-axis) chromatogram signals of Tris-blocked cross-linker bound to the asterisked cysteine sites of C*L (*m/z* = 597.4, z = +1), EGEEERLC*L (*m/z* = 721.1, z = +2), and C*LSPSPTSQRSRG (*m/z* = 869.7, *z* = +2) from in-gel chymotrypsin digestion of a coil 2 (286-400) R388C mutant protein, as described in the Materials and methods in the main text and supplementary Table 2.

c Tandem mass spectrum and proposed structure of a CL dipeptide bound to a Tris-blocked cross-linker.

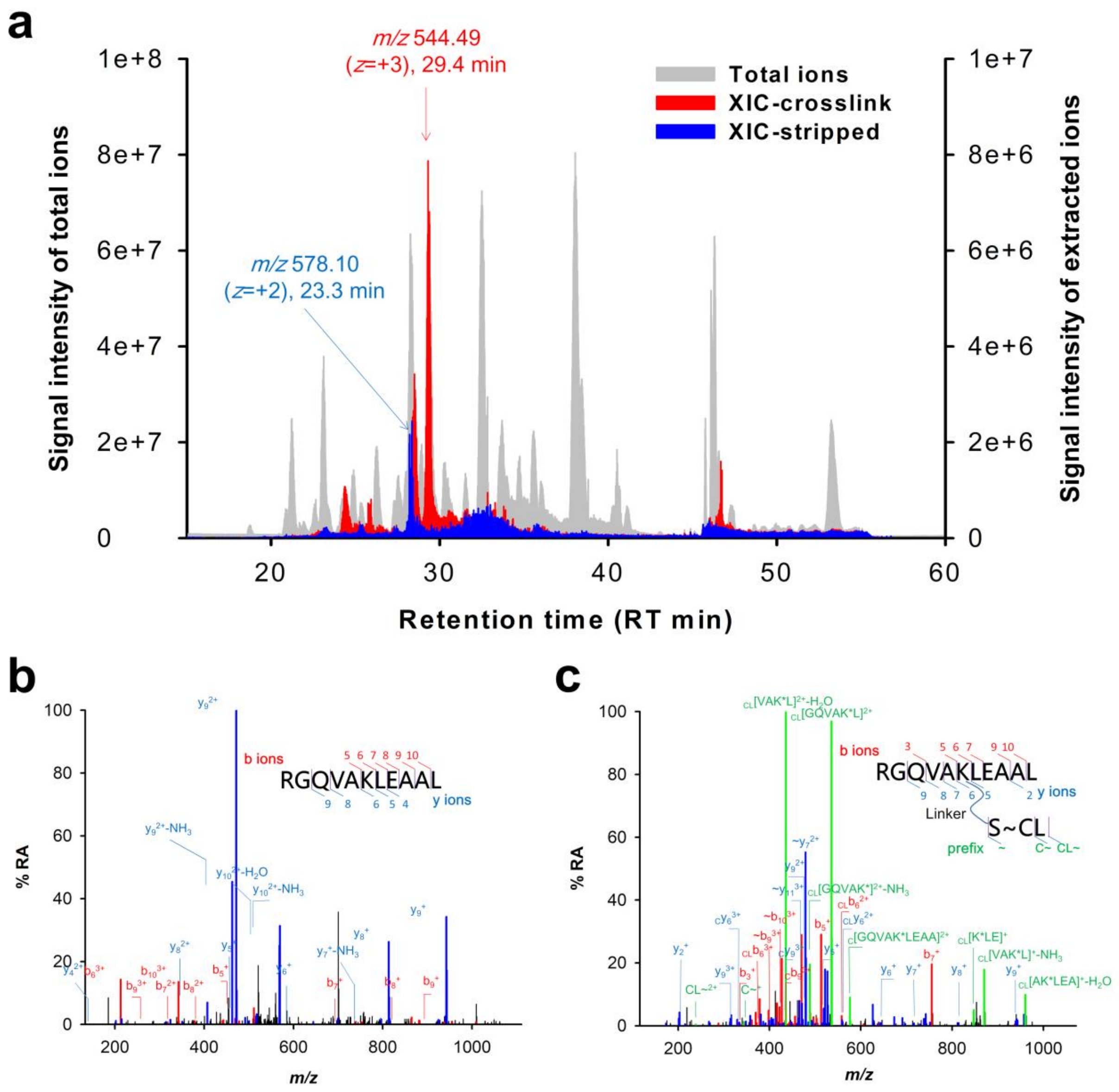

Supplementary Fig. 11. The most probable target peptide, ^{166}RGOVAK*LEAAL^{176}, in the lamin 300 fragment with or without modification of the asterisked Lsy171 position by a chemical cross-linker bound to a CL-dipeptide in the coil 2 286-400 R388C mutant protein

a Total ion (left y-axis) and extracted ion (right y-axis) chromatogram signals of the unmodified Lys171-containing peptide ion (stripped *m/z* = 578.1, *z* = +2) at the RT of 23.2 min and the CL-crosslinked Lys171-containing peptide ion (*m/z* 544.49, *z* = +3) at the RT of 29.4 min.

b An electrospray ionization-tandem mass spectrum of an unmodified Lys171-containing peptide.

c An electrospray ionization-tandem mass spectrum of a Lys171-containing peptide crosslinked with a CL dipeptide in the coil 2 286-400 R388C mutant protein.

References

1. Strelkov, S.V. & Burkhard, P. Analysis of alpha-helical coiled coils with the program TWISTER reveals a structural mechanism for stutter compensation. *J Struct Biol* **137**, 54-64 (2002).
2. Nicolet, S., Herrmann, H., Aebi, U. & Strelkov, S.V. Atomic structure of vimentin coil 2. *J Struct Biol* **170**, 369-376 (2010).
3. Chernyatina, A.A., Guzenko, D. & Strelkov, S.V. Intermediate filament structure: the bottom-up approach. *Curr Opin Cell Biol* **32**, 65-72 (2015).
4. Parry, D.A. Hendecad repeat in segment 2A and linker L2 of intermediate filament chains implies the possibility of a right-handed coiled-coil structure. *J Struct Biol* **155**, 370-374 (2006).
5. Aziz, A. *et al.* The structure of vimentin linker 1 and rod 1B domains characterized by site-directed spin-labeling electron paramagnetic resonance (SDSL-EPR) and X-ray crystallography. *J Biol Chem* **287**, 28349-28361 (2012).
6. Dittmer, T.A. & Misteli, T. The lamin protein family. *Genome biology* **12**, 222 (2011).
7. Szeverenyi, I. *et al.* The Human Intermediate Filament Database: comprehensive information on a gene family involved in many human diseases. *Human mutation* **29**, 351-360 (2008).
