# Supplementary material for: Structural basis for lamin assembly at the molecular level
Source: Nat Commun. 2019 Aug 21;10:3757. doi: 10.1038/s41467-019-11684-x (PMC6704074; doi:10.1038/s41467-019-11684-x)
Supplement: Supplementary file 3 — Description of Additional Supplementary Files [file 41467_2019_11684_MOESM3_ESM.pdf]

Description of Additional Supplementary Files

File name: Supplementary Data 1

Description: Tandem mass spectrometry analysis of lamin 300, coil 2 (286-400) R388C, and their crosslinked proteins.
